# Self-Perceived Interpersonal Problems Among Long-Term Unemployed Individuals, and Vocational Rehabilitation Programs (In)ability to Change Them

**DOI:** 10.1007/s10926-024-10188-w

**Published:** 2024-04-17

**Authors:** Martin Mau, Kirsten K. Roessler, Lotte N. Andersen, Maria L. Vang

**Affiliations:** 1 Department of Psychology, University of Southern Denmark, Odense, Denmark; 2 Health, Social Work and Welfare Research, UCL University College, Odense, Denmark; 3 Department of Sports Science and Clinical Biomechanics, University of Southern Denmark, Odense, Denmark; 4 Department of Occupational and Environmental Health, Odense University Hospital, Odense, Denmark

**Keywords:** Unemployment, rehabilitation, interpersonal problems

## Abstract

**Objective:**

Self-perceived interpersonal problems can challenge one’s access to the work market, making it harder to attain and keep a job while adding to the distress of being outside of the labor market.

**Methods:**

In this study, we compared the self-perceived interpersonal problems among long-term unemployed individuals taking part in vocational rehabilitation programs (VRPs) (*N* = 220) with those of the general population. In addition, we examined whether their self-perceived interpersonal problems changed while taking part in the VRPs.

**Results:**

We found that participants report significantly higher levels of self-perceived interpersonal problems as measured by the Inventory of Interpersonal Problems (IIP), especially with regard to feeling cold/distanced, socially inhibited, vindictive/self-centered, and non-assertive. The participants did not report a significant decrease in self-perceived interpersonal problems after being part of VRPs for one year.

**Conclusion:**

These results are relevant as they may inform interventions targeted this population aimed at increasing employability and/or individual well-being. Importantly, the findings may be viewed as a reflection of both social and individual processes. Long-term unemployed individuals’ tendency to feel insufficiently engaged may reflect difficulty with keeping up with a job market in constant change.

**Supplementary Information:**

The online version contains supplementary material available at 10.1007/s10926-024-10188-w.

## Introduction

Employment and a stable connection to the labor market are important to individuals’ psychological health and well-being [1]. For many, work is an important source of identity, sense of meaning and purpose in life, and of social connection [2, 3]. Conversely, individuals who are *outside* of the labor market – e.g. people who are experiencing long-term unemployment – have an increased risk of poor mental health [4, 5]. In addition to being problematic in itself, mental health challenges may both be a cause and effect of unemployment. People with mental health challenges may be perceived to be less fit for work than others, reducing their (perceived) employability (Brohan et al., 2012[^32^]). On the other hand, being long-term unemployed or losing one’s job can lead to psychological distress [4, 6, 7]. Indeed, the recent Danish Health Profile indicated that the individuals reporting the highest levels of stress were those who were unemployed [8].

Vocational rehabilitation programs (VRP) are used to increase the employability of individuals with social and health-related problems,. Through an individually tailored set of activities administered by a municipal interdisciplinary rehabilitation team, the aim is to help people prepare to or regain the ability to join the workforce [9, 10]. When considering the effects of such programs, it is important to extend the focus beyond whether or not participants actually join the workforce and gain employment. Research into VRPs regarding ‘what works’ will need to include ‘effects’ or consequences other than the narrow objective of labor market participation when examining ‘what works’” [11], p. 15–16). Such measures may not be related to what benefits society economically, but also whether they enable participants to live a more engaged and gratifying life.

This article will follow this recommendation by focusing on VRPs and their influence on an aspect believed to be fundamental to mental health, namely self-perceived interpersonal problems [12, 13]. Self-perceived interpersonal problems are relational issues that the individual perceives to be having, for example finding it difficult to join groups, letting one’s needs known to others, or expressing affection [14]. According to a psychodynamic and object relations theoretical perspective [15, 16], self-perceived interpersonal problems can be described along two axes. The first axis regards *affiliation* as a matter of perceiving oneself to be too cold or too nurturing. The second axis regards *power*. On the one end of this axis, the person perceives oneself to be too dominating, on the other, non-assertive [17]. This measure of self-perceived interpersonal problems has been used in other studies in connection with, e.g. alcohol abuse [18], and long-term illness [19].

In addition to indicators of health problems such as alcohol abuse and long-term illness, self-perceived interpersonal problems are also associated with work-related factors such as lower job involvement and satisfaction, and increased occupational stress [20, 21]. Additionally, self-perceived interpersonal problems may affect well-being [22] and are in themselves cause for distress. Furthermore, identifying which interpersonal issues that this group is experiencing could inform VRPs, potentially improving the psychological and social gains of such programs. Although research suggests that unemployed individuals do not actually have lower social and communicative competencies [23], examining if and how unemployed individuals *perceive* themselves to be interpersonally challenged is therefore still relevant.

### Aim

The aim of this study is twofold: 1) to compare the self-perceived interpersonal problems among individuals who are unemployed with social and health-related problems participating in a VRP, to those of a general population norm, and 2) to examine whether self-perceived interpersonal problems change after participation in the municipal VRP.

## Methods

### Study Design

This study was a longitudinal survey, where participants’ self-perceived interpersonal problems were measured at baseline and at one-year follow-up. The baseline measurement was conducted by sending out a questionnaire by letter to all individuals, enrolled in a VRP within the last year in one Municipality in Denmark. One year later, the same questionnaire was sent out to the individuals who had responded to the first questionnaire. In case participants did not respond to the questionnaire the first time it was sent out, and a telephone call was made after two weeks to remind them. After four weeks, the questionnaire was sent out again to those who did not respond the first time. This study was part of a larger intervention, and additional details are described elsewhere [9, 10].

### Participants

Participants (*N* = 220) in this study were all enrolled in a VRP in a municipality in Denmark. To become enrolled in the VRP, the following criteria, as defined by the Danish Ministry of Employment, were applied: Being between 18–65 years and unemployed; having a high risk of being placed on permanent disability pension due to health and/or social problems affecting employability; needing continued education or retraining of skills; not being ready to enter the labor market. There were no diagnostic criteria for being included in a VRP. Participation was decided not on the basis of what specific types of illnesses, but whether participants fulfilled the criteria stated above.

### Intervention: Vocational Rehabilitation Program (VRP)

As explained earlier, VRPs consist of an individually tailored set of activities, administered by an interdisciplinary rehabilitation team. Both content and duration are adjusted to the individual participant. It may last one to five years, and include one or several of the following: Work and/or social skills training (such as internship programs), self-management courses related to mental or physical health (for example focusing on anxiety or overweight), provision of support services from the municipality (for example through health counseling or a supportive contact person), rehabilitation activities (for example physiotherapy), and educational services. A citizen’s eligibility to participate in a VRP is decided by a social worker at a job center. Whether to participate in the program is then decided by an interdisciplinary rehabilitation team, in consultation with the citizen. In case a participant wished to discontinue with the program (and thus drop out of the study), there were no consequences of this. If the person chose to discontinue the program, they would transfer to other parts of the social system.

### Outcome: Inventory of Interpersonal Problems (IIP-64)

Self-perceived interpersonal problems are assessed using the questionnaire Inventory of Interpersonal Problems (IIP) [16] which builds on psychodynamic and object relations theory. The 64-item version of The Inventory of Interpersonal Problems was used to assess self-perceived interpersonal problems [16]. The IIP-64 comprises eight subscales, each with eight items, describing some particular domain of interpersonal distress. These subscales express a combination of the two main axes, affiliation and power.

At each end of these two main axes is a subscale (yielding four subscales). On the power axes, the first subscale is *dominance*, which regards the perception that one is too controlling. The subscale at the opposite end is *non-assertive,* which regards having problems making one’s needs known. On the affiliation axes, the first subscale is *excessively nurturant*, which regards being too caring and trusting. The subscale at the opposite end is *cold*, which regards problems related to making long-term commitments and expressing affection [17].

**Fig. 1:**
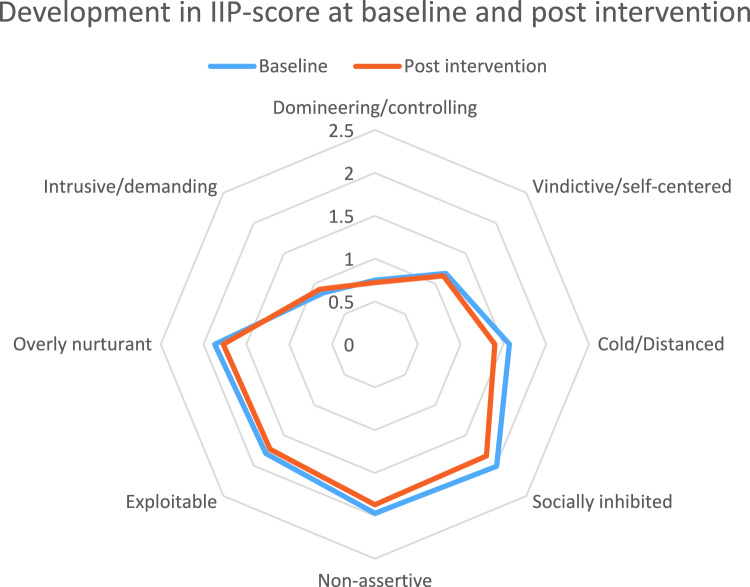
Radar diagram illustrating IIP-score across baseline and post intervention

The two main axes are placed orthogonally in a circumplex model, and in between them, there are an additional four subscales [17]. These subscales are: *Intrusive*, which for example regards perceiving oneself as being too self-disclosing; *exploitable*, which regards being too easily taken advantage of and difficulty expressing anger; *socially inhibited*, which regards, for example, problems related to approaching others; and finally, *vindictive*, which regards problems related to caring about needs of and trusting, others [17] (A graphic illustration of the IIP-model can be seen in Fig. 1).

The IIP is scored on a 5-point Likert-like scale ranging from 0 (‘not at all’) to 4 (‘extremely’). A higher score indicates increasing difficulty regarding the specific interpersonal situation the item relates to [17]. Research supports both the construct validity test–retest reliability and internal consistency of the IIP [16, 24]. The IIP can be used to assess the general level of self-perceived interpersonal problems, and/or self-perceived interpersonal problems on the individual subscales.

### Statistical Analysis

The range and distribution of socio-demographic variables at baseline were calculated using descriptive statistics. For comparing self-perceived interpersonal problems at baseline between those participating in the intervention and a general population (research question 1), one sample t-test was conducted. General population mean scores were obtained via personal communication with the publisher (Roessler, personal communication, 2016). For assessing change in perceived interpersonal problems across the intervention (research question 2), we performed a series of dependent samples t-test for each of the eight IIP subscales and IIP total. For all analyses, 95% CI’s and p-values were calculated with a bootstrap process using 1000 draws using the bias corrected accelerated (Bca) approach. Cases were excluded on a case-wise basis and analyses were conducted using SPSS version 28.0.1.0. Missing data on individual items on the IIP ranged from 2.1% to 6.2%.

## Results

### Demography

Table 1 displays descriptive statistics of the sample. Overall, the majority of participants were women, and the majority had some form of vocational education. Most participants were Danish and did not have any live-in children. Approximately half were in a cohabiting relationship. Additionally, with regards to the drop outs of the follow-up measure in the study, there was no age-differences (t(98.07) = 0.42, p = 0.680), gender differences (chi2(1) = 0.005, *p* = 0.942), differences related to having live-in children or not (chi2(1) = 2.52, *p* = 0.113), or whether participants lived with a partner or not (chi2(1) = 0.00, *p* = 1.00).

**Table 1:** Sample characteristics at baseline

Gender (women, N, %)	155	70.5%
Age (M, SD)	42.65	11.30
No vocational education (N, %)	79	35.9%
Marital status (living with partner, N, %)	107	48.6%
Parental status (live-in children, N, %)	80	36.4%
Country of origin (Other than Denmark, N, %)	45	20.5%

### IIP Profile of Participants, Compared to a General Population Norm

At baseline, the participants in this study had significantly higher scores on all subscales of the IIP compared to a (Danish) general population norm (see Table 2). The largest difference was found on the subscales relating to the cold/distanced subscale, and the socially inhibited subscale, followed by the vindictive/self-centered subscale and the non-assertive subscale.

**Table 2:** Comparison of IIP-scores across baseline and general population norms

	N	Baseline		Compared with the general population norm, One sample t-test, bootstrapped p
	N	M	SD	
Domineering/controlling	131	0.82	0.60	*p* < 0.001
Vindictive/self-centered	132	1.29	0.84	*p* <0 .001
Cold/Distanced	135	1.56	0.97	*p* < 0.001
Socially inhibited	136	1.80	0.98	*p* < 0.001
Non-assertive	132	1.84	0.93	*p* < 0.001
Exploitable	131	1.74	0.77	*p* < 0.001
Overly nurturant	132	1.88	0.83	*p* < 0.001
Intrusive/demanding	132	0.99	0.59	*p* < 0.001

Note: Danish population norms are retrieved from Hogrefe via personal communication (Roessler, 2016). Exact *t*-values are omitted to protect copyrighted norms. Bootstrap is conducted with 1000 samples

### Development in Self-Reported Interpersonal Problems Before and After Intervention

Figure 1 and Table 3 display findings from the paired samples t-test. Only participants with full response profiles were included in the paired samples t-test, leaving an active sample of *n* = 51 for comparison. Independent samples t-test indicated that there were no statistically significant differences in baseline-scores between participants who responded at follow-up and those who did not (*p*-values ranging between 0.065 to 0.840 for social inhibition and vindictive, respectively). Before correction for multiple testing, there were no statistically significant differences between baseline and follow-up scores on either subscale. Hence, no corrections were made as there were no statistically significant findings to ensure the robustness of via correction for multiple testing.

**Table 3:** Comparison of IIP-scores across baseline and post intervention

	N	Baseline		Follow up		Dependent samples *t*-test, 95% CI, *p*
	N	M	SD	M	SD	
Domineering/controlling	51	0.75	0.57	0.72	0.68	*t*(50) = 0.38, [−0.12;0.17], *p* = 0.707
Vindictive/self-centered	51	1.17	0.92	1.13	0.83	*t*(50) = 0.50, [−0.12;0.19], *p* = 0.616
Cold/Distanced	51	1.57	1.07	1.40	0.90	*t*(50) = 1.44, [−0.07;0.39], *p* = 0.157
Socially inhibited	51	2.01	1.10	1.84	1.04	*t*(50) = 1.47, [−0.06;0.41], *p* = 0.147
Non-assertive	51	1.97	1.06	1.87	1.00	*t*(50) = 0.84, [−0.13;0.32], *p* = 0.403
Exploitable	51	1.80	0.92	1.73	0.89	t(50) = 0.64, [−0.15;0.30], *p* = 0.528
Overly nurturant	51	1.87	0.83	1.77	0.83	*t*(50) = 1.26, [−0.06;0.27], *p* = 0.212
Intrusive/demanding	51	0.85	0.55	0.91	0.58	*t*(50) = −0.89, [−0.22;0.08], *p* = 0.380

## Discussion

This study had two aims, the first regarding which interpersonal problems characterized long-term unemployed individuals taking part in VRPs when compared to a general population norm, and the second regarding the change in self-perceived interpersonal problems after participation in VRPs.

The answer to the study’s first aim is that the participants scored significantly higher on all IIP-subscales compared to the general population, meaning that the participants in the VRPs generally perceive themselves to have more interpersonal problems than reported in the general population. Especially on the subscales of cold/distanced, socially inhibited, vindictive/self-centered, and non-assertive, the participants in this study achieved a higher score. Scoring higher on these subscales indicates that participants generally perceive themselves to have problems with approaching others, expressing affection, and caring about the needs of others, while also making their own needs known [17].

Compared to other research on social traits viewed as desirable in the labor market, such perceptions may be especially challenging. Following the five-factor model, research suggests that traits associated with extraversion, including sociability, dominance, and positive emotions are generally valued in the labor market [25]. Findings from this study, e.g. that unemployed individuals tend to feel overly cold towards others and feel they have problems approaching others and making their needs known, can be seen as close to the opposite of this. Although this study did not examine personality traits as such, and cannot, therefore, be directly compared to these findings, these findings indicate a lower prevalence of desired interpersonal qualities in the labor market among unemployed individuals (and self-perceived interpersonal problems also can also be related to fundamental aspects of the individual’s psychology, e.g. attachment styles (Haggerty et al., 2009[^33^])).

Importantly, however, as this study examines *perceptions* among unemployed individuals, this overall more positive view on extraversion in the labor market is also a *perception*. This perception may be partially misplaced as suggested by research indicating that workplace *diversity* is associated with higher productivity [26]. Therefore, it may be discussed whether a change in individuals’ interpersonal profiles is warranted, more than a change in perceptions of what makes employees desirable in the labor market. The implication for the VRPs in light of this study could be to pay attention to both. However, as self-perceived interpersonal problems are unwarranted in themselves – not only for the labor market but also for the individual, it is relevant to address this issue independent of the possible effect on employability. Moreover, that the participants described having more interpersonal problems can be viewed as a consequence of problems that transcend both individuals and individual workplaces, but rather relate to broader societal issues. Thus, one discussion that can be raised on the basis of this study, regards what lies behind these findings. Although self-perceived interpersonal problems exists, at some level, within the individual, the causes may mirror society more broadly.

According to Hartmut Rosa’s theory on social acceleration, modern society (and its’ workplaces) are in a state of “frenetic standstill” (Rosa, 2013^34^). On the one hand, nothing remains stable; change is considered so desirable, that it is happening *frenetically*. This change, however, is only at the surface level, whereas on a deeper level, and contrary to ambition, nothing essentially changes.

The perception among the unemployed that they were having more interpersonal problems can be viewed as a consequence of trying to accommodate this demand for constant change. Feeling insufficiently engaged (not expressing own needs enough, or caring about the needs of others), may be a reaction to trying to keep up with a workplace, which is always in flux. Thus, self-perceived interpersonal problems may be felt and expressed at the individual level, but actually stem from broader issues.

Regarding the study’s second aim, the result showed that the self-perceived interpersonal problems did not change during the course of VRPs. On neither of the IIP-subscales did the intervention group achieve a statistically significant improvement. This may illustrate the limits of the VRPs offered to the long-term unemployed. Self-perceived interpersonal problems may be too fundamental to be changeable within the time- and resource-related limits of VRP and may instead be more suitable targets for a psychotherapeutic intervention [15, 21]. Nevertheless, the so-called soft skills associated with employability (see e.g., [27]), for example one’s approach to interpersonal interactions, has been examined in other studies. According to these, soft skills, although perhaps difficult to develop [28], can be essential for people entering the labor market [29, 30]. Again, however, this may more aptly be the subject of psychotherapy, rather than VRPs as they are currently designed. An alternative interpretation might be that the 1-year interval between measurements does not capture the potential changes in self-perceived interpersonal problems during the course of a VRP as the interventions individually tailored and last between 1 to 5 years. Methodological limitations in the current design preclude any causal inferences regarding the impact of VRPs on self-perceived interpersonal problems.

Self-perceived interpersonal problems have been found challenging to reduce in earlier studies [18, 19]. Also, this finding does not necessarily entail that VRPs should be considered unsuccessful, as they may have other effects. It does, however, call into question if the VRPs are sufficiently adapted to mental health challenges among the unemployed, which the programs are designed to intervene against. The finding in this study echoes our earlier publications on the negligible effects of VRPs on other measures of psychological health, including well-being [10]. This does not necessarily entail, however, that VRPs are irrelevant. Rather, as suggested in the introduction of this article, research into VRPs ought to continue to explore different outcomes and different interpretations of these. The above-mentioned interpretation of self-perceived interpersonal problems in light of Rosa’s theory of social acceleration further questions whether VRPs are in a position where they can counter challenging dynamics in society more broadly.

### Limitations

When interpreting these findings, it is important to take into account certain methodological considerations. First, the study was nested in a municipality-based practice and was not a research-based RCT including a control group. Regarding the intervention, VRPs are challenging to study. A VRP can take from 1–5 years, but in this study, self-perceived interpersonal problems were only measured at baseline and one-year follow-up. Thus, we may not have captured the full effect of the intervention, and a longer longitudinal study, with more assessment points, could be useful. Relatedly, the content of VRPs is individually tailored. Perhaps parts of the VRPs do address self-perceived interpersonal problems. Pooling all aspects of the VRPs together may therefore limit the findings. Aspects of the intervention could have provided an effect, but since VRPs examined here may vary in both duration and content, such an effect may have been buried among ineffective components. Moreover, the non-response and drop-out rates for this study were quite high. This may have impacted the study’s ability to answer the research questions. However, there were no statistically significant differences on demographic factors or the outcome measures between dropouts and completers of the follow-up survey, and therefore, any systematic differences between dropout and completers would be related to factors beyond the scope of the current study.

## Conclusions

The long-term unemployed participating in this study reported significantly more self-perceived interpersonal problems than the general population, especially with regards to feeling cold/distanced, socially inhibited, vindictive/self-centered, and non-assertive. The self-perceived interpersonal problems did not decline significantly over the course of one year with vocational rehabilitation. This may point to the limits of the VRPs to influence mental health challenges among the long-term unemployed; more thorough, psychotherapeutic interventions may be needed. Although VRPs already have components targeted the training of social skills and courses in self-management, additional components of the programs focusing on thoughts and feelings surrounding social interaction at work, could be beneficial.

Viewed in light of labor market preferences for extraverted individuals, the interpersonal problems identified in this study can be challenging. Addressing both the perceptions among the long-term unemployed can be relevant, as can workplace values.

## Supplementary Information

Below is the link to the electronic supplementary material.

Supplementary file1 (DOCX 17 KB)

## Data Availability

The datasets that we generated and analyzed during the current study is not publicly accessible in order to preserve the privacy of participants.
