# Supplementary material for: Self-Perceived Interpersonal Problems Among Long-Term Unemployed Individuals, and Vocational Rehabilitation Programs (In)ability to Change Them
Source: J Occup Rehabil. 2024 Apr 17;35(1):88–95. doi: 10.1007/s10926-024-10188-w (PMC11839694; doi:10.1007/s10926-024-10188-w)
Supplement: Supplementary file 1 — Supplementary file1 (DOCX 17 KB) [file 10926_2024_10188_MOESM1_ESM.docx]

Supplementary table 1: Scores on IIP at baseline and 1 year follow-up for full sample

|  | Baseline | | | Follow up | | |
| --- | --- | --- | --- | --- | --- | --- |
|  | N | M | SD | N | M | SD |
| Domineering/controlling | 131 | 0.82 | 0.60 | 67 | 0.77 | 0.67 |
| Vindictive/self-centered | 132 | 1.29 | 0.84 | 67 | 1.18 | 0.80 |
| Cold/Distanced | 135 | 1.56 | 0.97 | 66 | 1.45 | 0.85 |
| Socially inhibited | 136 | 1.80 | 0.98 | 66 | 1.75 | 0.98 |
| Non-assertive | 132 | 1.84 | 0.93 | 63 | 1.88 | 0.95 |
| Exploitable | 131 | 1.74 | 0.77 | 64 | 1.78 | 0.84 |
| Overly nurturant | 132 | 1.88 | 0.83 | 66 | 1.82 | 0.79 |
| Intrusive/demanding | 132 | 0.99 | 0.59 | 67 | 0.99 | 0.61 |
